# The Impact on Infectivity and Neutralization Efficiency of SARS-CoV-2 Lineage B.1.351 Pseudovirus

**DOI:** 10.3390/v13040633

**Published:** 2021-04-07

**Authors:** Yeong Jun Kim, Ui Soon Jang, Sandrine M. Soh, Joo-Youn Lee, Hye-Ra Lee

**Affiliations:** 1 Department of Biotechnology and Bioinformatics, College of Science and Technology, Korea University, Sejong 30019, Korea; kyj1994@korea.ac.kr (Y.J.K.); jus119@korea.ac.kr (U.S.J.); sandy319@korea.ac.kr (S.M.S.); 2 Therapeutics & Biotechnology Division, Korea Research Institute of Chemical Technology, 141 Gajeong-ro, Yuseong-gu, Daejeon 34114, Korea; leejy@krict.re.kr; 3 Department of Lab Medicine, College of Medicine, Korea University, Seoul 02841, Korea

**Keywords:** SARS-CoV-2, B.1.351, K417N and E484K mutation of spike, viral infectivity, neutralization, Casirivimab, Imdevimab

## Abstract

A new variant of SARS-CoV-2 B.1.351 lineage (first found in South Africa) has been raising global concern due to its harboring of multiple mutations in the spike that potentially increase transmissibility and yield resistance to neutralizing antibodies. We here tested infectivity and neutralization efficiency of SARS-CoV-2 spike pseudoviruses bearing particular mutations of the receptor-binding domain (RBD) derived either from the Wuhan strains (referred to as D614G or with other sites) or the B.1.351 lineage (referred to as N501Y, K417N, and E484K). The three different pseudoviruses B.1.351 lineage related significantly increased infectivity compared with other mutants that indicated Wuhan strains. Interestingly, K417N and E484K mutations dramatically enhanced cell–cell fusion than N501Y even though their infectivity were similar, suggesting that K417N and E484K mutations harboring SARS-CoV-2 variant might be more transmissible than N501Y mutation containing SARS-CoV-2 variant. We also investigated the efficacy of two different monoclonal antibodies, Casirivimab and Imdevimab that neutralized SARS-CoV-2, against several kinds of pseudoviruses which indicated Wuhan or B.1.351 lineage. Remarkably, Imdevimab effectively neutralized B.1.351 lineage pseudoviruses containing N501Y, K417N, and E484K mutations, while Casirivimab partially affected them. Overall, our results underscore the importance of B.1.351 lineage SARS-CoV-2 in the viral spread and its implication for antibody efficacy.

## 1. Introduction

The COVID-19 is an ongoing pandemic caused by severe acute respiratory syndrome coronavirus 2 (SARS-CoV-2). This COVID-19 pandemic has occurred not only as a devastating global health crisis with notable mortality but also tremendous socioeconomic damage. As of 6 February 2021, more than 100 million confirmed cases and 2 million deaths have been reported globally. Thus, in an attempt to put an end to this pandemic, one of the encouraging approaches is the development of anti-viral antibodies that neutralize spike protein of SARS-CoV-2. The spike protein of SARS-CoV-2 has been shown to be necessary for viral entry into the host cells. For this, the S1 subunit of spike protein binds to the cellular receptor, angiotensin-converting enzyme 2 (ACE2) and the S2 subunit of spike promotes fusion between viral and cellular membranes. In addition, the spike glycoprotein being a key antigenic determinant, serves as a primary target of neutralizing antibodies. Because of the two functions mediated by the spike protein, mutations of this protein are quite major concerns, as they alter the biochemistry of the spike, which in turn affects the transmissibility, and/or immunogenicity of SARS-CoV-2.

In fact, SARS-CoV-2 continues to accrue considerable mutations in its genome despite harboring the proofreading function [1]. The first reported emerging SARS-CoV-2 mutation, D614G, has now become the most common form in the world. Mounting results reported that pseudoviruses encoding D614G mutant show higher infectivity than the original strain [2,3,4]. A SARS-CoV-2 variant with D614G substitution was shown to enhance transmissibility and competitive fitness in animal models and human cell cultures [4]. Furthermore, structural analysis elucidated that D614G mutation led to a conformational change of the receptor-binding domains (RBDs), indicating that enhanced the ability to attach to the ACE2, ultimately induced higher infectivity [5,6]. The patients who were infected with SARS-CoV-2 D614G strain were associated with higher viral loads but not disease severity and mortality [7]. Fortunately, vaccines with verified efficacy against the ancestral SARS-CoV-2 are also efficacious against the D614G variant [8]. 

Recently, the new emergence of SARS-CoV-2 variants B.1.1.7 in the UK and B.1.351 in South Africa (SA) have raised particular concerns owing to increased prevalence and multiple mutations in the spike protein [9]. In the case of B.1.1.7, a series of mutations in eight sites including D614G appeared in the spike protein: Δ69Δ70, Δ144Δ145, N501Y, A570D, P681H, T716I, S982A, and D1118H (https://www.gisaid.org; GISAID, accessed on 1 December 2020). Among them, Δ69Δ70 and N501Y in B.1.1.7 lineage have been shown to cause a conformational change in the spike protein and have higher infectivity than D614G, suggesting that they may increase transmissibility and alter antigenicity [10,11]. Remarkably, B.1.351 contains additional two mutations, K417N and E484K, in receptor binding motif (RBM) of the RBD region and those may potentially induce a conformational change of the spike protein, and subsequently increase the infectivity of B.1.351 than other lineages. However, it is still unclear as to whether those mutations may affect viral infectivity, transmissibility, or antibody-mediated neutralization.

In this study, we examined the biological significance of SARS-CoV-2 variant B.1.351 with particular amino acid changes in the spike protein. To address this, we generated seven different spike mutants in RBD that indicated either Wuhan or B.1.351 and then analyzed their infectivity, cleavage efficacy, and escape capacity to neutralizing antibodies using pseudoviruses. We show that SARS-CoV-2 B.1.351 variant harboring K417N and E484K mutations have naturally evolved to possess notable alterations in their infectivity and S1/S2 cleavage, leading to enhanced syncytium formation and antigenicity. Fortunately, we found that Imdevimab was still able to fully neutralize against the B.1.351 lineage, while Casirivimab significantly reduced its neutralizing activity.

## 2. Materials and Methods

### 2.1. Cell Culture and Cell Line Construction

293T, 293T-ACE2, and Vero cells were cultured in Dulbecco’s modified Eagle’s medium (DMEM) supplemented with 10% fetal bovine serum (FBS) and 1% penicillin-streptomycin (P/S) (Hyclone). Caco-2 and Calu-3 cells were maintained in minimum essential medium (MEM) supplemented with 10% FBS and 1% P/S. To generate 293T cell line stably expressing ACE2, 293T cells were transfected with pCEP4-myc-hACE2 (addgene, Watertown, MA, USA) using PEI (Sigma, Munich, Germany). Transfected cells were selected with 100 μg/mL of hygromycin B (Invitrogen, Waltham, MA, USA) at 48 h after transfection. The hygromycin B-resistant 293T-hACE2 cell lines were established after 2 weeks of hygromycin B selection.

### 2.2. Plasmid Construction

The coding sequence of SARS-CoV-2 Spike was amplified from pUC57- SARS-CoV-2 plasmid (synthesized by Bionics, Seoul, Republic of Korea) via PCR and subcloned into the pCMV vector at the restriction sites NheI and BamHI. Subsequently, SARS-CoV-2 Spike variants (D614G, V367F/D614G, P384L/D614G, R408I/D614G, N501Y/D614G, K417N/N501Y/D614G, E484K/N501Y/D614G) were generated using a Quikchange XL site-directed mutagenesis kit (Agilent, Santa Clara, CA, USA) according to manufacturer’s instructions. The primers used to generate SARS-CoV-2 Spike variants are described as follow: V367F forward 5′-GCTGACTACTCTTTCCTCTACAACTCT-3′; V367F reverse 5′-AGAGTTGTA GAGGAAAGAGTAGTCAGC-3′; P384L forward 5′-ATGGAGTGAGCC TAACCAAACT GAA-3′; P384L reverse 5′-TTCAGTTTGGTTAGGCTCACTCCAT-3′; R408I forward 5′-GAGATGAGGTGATACAGATTGC CCC-3′; R408I reverse 5′-GGGGCAATCTGTATCACCTCATCTC-3′; K417N forward 5′-GACAAACAGGCAACATTGCT GACTACA-3′; K417N reverse 5′-TGTAGTCAGCAATGTTGCCTGTTTGTC-3′; E484K forward 5′-ATG AATGGAGTGAAGGGCTTCAACTG-3′; E484K reverse 5′-CAGTTGAAGCCCTTCACT CCATTACAT-3′; N501Y forward 5′-CTTCCAACCAACCTATGGAGTG GGCTA-3′; N501Y reverse 5′-TAGCCCACTCCATAGGTTGGTTGGAAG-3′; D614G forward 5′-TGC TCTACCAGGGTGTGAACTGTAC-3′; D614G reverse 5′-GTACAGTTCAC ACCCTGGTAGAGCA-3′.

### 2.3. Chemicals and Antibodies

Chemicals were purchased from the following manufacturers: Bright-Glo, FuGENE HD transfection reagent from Promega, Madison, WI, USA; PEI from Sigma; TRIZOL and TPCK-treated trypsin from Thermo Fisher, Waltham, MA, USA; SYBR Green from Bio-Rad, Hercules, CA, USA. Antibodies targeting SARS-CoV-2 Spike (GTX632604) was obtained from Genetex, Irvine, CA, USA. Anti-β-actin antibody (A5316) was purchased from Sigma.

### 2.4. Pseudo-Type Virus Production and Titration

Replication-deficient retrovirus-based different pseudoviruses bearing SARS-CoV-2 spike wild-type (WT) or several mutations were generated by following a procedure previously described [12]. Briefly, 293T cells were transfected with plasmids encoding murine leukemia virus (MLV) gag/pol, individual SARS-CoV-2 spike variants and firefly luciferase reporter. Forty-eight hours post-transfection, pseudoviruses containing culture supernatants were harvested, filtered (0.45-μm pore size; Merck Millipore, Burlington, MA, USA) and stored at −80 °C until use.

The titer of generated pseudoviruses was measured by quantitative RT-PCR. Total RNA from the supernatant was extracted using TRIZOL (Thermo Fisher) following the manufacturer’s instruction. Subsequently, cDNA was synthesized with cDNA synthesis kit (Toyobo, Osaka, Japan) and real-time PCR was performed using SYBR-green and CFX96 Real-Time System (Bio-rad). The primer pair targeting MLV 3′ LTR used to detect pQCXIP- firefly Luciferase (pQCXIP-fLuc) reporter plasmid are described below [13]: Forward 5′-ATTGACTGAGTCGCCCGG-3′; Reverse 5′-AGCGAGACCACAAGTCGGAT-3′. The copies of reporter plasmid from each pseudotype viral supernatant were calculated based on the pQCXIP-fLuc plasmid standard curve.

### 2.5. Measurement of Infectivity

Using the quantitative RT-PCR, we calculated RNA copy numbers and normalized the pseudovirus to the same amount. After normalization, individual spike variant pseudoviruses or VSV-G pseudovirus were transduced into different cell types and added Bright-Glo (Promega) at 24 h after transduction. Relative luminescence unit (RLU) was measured by Varioskan Lux microplate reader (Thermo). Results were calculated as a folding activity compared to the SARS-CoV-2 spike wild-type sample. All experiments were performed in triplicate.

### 2.6. Spike Cleavage Assay

Briefly, the same titer of pseudovirus expressing spike variants or VSV-G was transduced into three different cell lines (Vero, Caco-2, and Calu-3). At 24 h post-transfection, the cells were harvested with a cell scraper and performed immunoblotting using SARS-CoV-2 spike antibody (Genetex) targeting spike S2 domain. The band intensity of full-length spike and cleaved S2 domain of spike was analyzed by EvolutionCapt software (Vilber Lourmat, Collegien, France) and the ratio of cleaved S2 domain was calculated.

### 2.7. Membrane Fusion Assay

Plasmids encoding SARS-CoV-2 spike WT or variants were transfected into Vero cells using FuGENE HD (Promega) following the protocol provided by the manufacturer. Twenty-four hours after transfection, cells were treated with 1 μg/mL of TPCK-trypsin (Thermo) for 6 h and the medium was replaced with complete DMEM. Then cells were incubated for 18 h and fixed with 4% paraformaldehyde followed by Giemsa staining. Stained cells were visualized with EVOS-M5000 imaging system (Invitrogen) and syncytia containing more than 3 nuclei were counted. 

### 2.8. Pseudovirus-Based Neutralization Assay

Herein, known neutralizing monoclonal antibodies for SARS-CoV-2, Casirivimab and Imdevimab, were chosen for neutralization assay [14,15]. In brief, receptor-binding domain (RBD) of the spike was synthesized and cloned into an inline fusion of C-terminus Fc tagged vector for mammalian cell expression in a form of RBD-Fc [14]. The construct was transfected into HEK293E cells and protein G agarose (Merck Millipore) was used for affinity purification of the expressed RBD-Fc. The efficacy of neutralization was measured by the reduction of luciferase expression, as described previously [12]. In brief, individual pseudoviruses were incubated with serial dilutions of monoclonal antibody preparation for 30 min at room temperature, as triplicate and subsequently was added to the cells. Following 24 h of incubation, the luminescence was measured by Varioskan Lux microplate reader. At least triplicate results were obtained. The IC_50_ values were calculated with non-linear regression, i.e., log (inhibitor) vs response (four parameters), using GraphPad Prism 8 (GraphPad Software, Inc., San Diego, CA, USA). 

### 2.9. Statistics

Statistical analyses were performed with GraphPad Prism 8 software. *p*-values were calculated by Student’s t-test and *p* < 0.05 denoted statistical significance. Data are presented as the mean ± SD.

### 2.10. Structure Preparation

The structures used to model the wild-type trimeric spike protein of SARS-CoV-2 and human ACE2 protein were taken from the PDB site (https://www.rcsb.org, accessed on 1 December 2020), which is determined by cryo-electron microscopy (cryo-EM) of SARS-CoV-2 spike in complex with an antibody (PDB code 72CL) and of full-length human ACE2 in complex with SARS-CoV-2 spike RBD (PDB code 6M17). In order to build missing residues in the spike protein, homology modeling was employed to generate the whole spike protein structure using the SWISS-MODEL server (https://swissmodel.expasy.org, accessed on 1 December 2020, accession number P0DTC2). The 4 selected mutants (D614G, N501Y/D614G, K417N/501Y/D614G, and E484K/N501Y/D614G) in the spike protein were generated using the Build Mutant protocol in Discovery Studio 2020 (Dassault Systèmes BIOVIA, San Diego, CA, USA, 2020). The complex structures between the SARS-CoV-2 spike protein and ACE2 were constructed based on the cryo-EM structures as a template using the Superimpose Proteins Tool in Discovery Studio 2020.

### 2.11. Molecular Dynamics (MD) Simulation

The monomeric SARS-CoV-2 spike/ACE2 residues 21-615 complex models for each mutant were used as the starting structures in the MD simulations. Desmond v6.4 (Desmond Molecular Dynamics System; D. E. Shaw Research: New York, NY) and OPLS3e force field were used in the MD simulations using Schrödinger Suite 2020-4 (Schrödinger, LLC, New York, NY, USA, 2020). The System Builder was used for solvation employing predefined TIP3P water molecules in an orthorhombic box with dimensions of 15 Å × 15 Å × 15 Å, and the overall complex was neutralized by adding Cl^−^ counterions. The NaCl salt concentration was 0.15 mol/L. Production of MD simulations at 100 ns in length proceeded under periodic boundary conditions in the NPT ensemble at normal temperatures (300 K) and pressure (1.01325 bar), where the coordinates were written at intervals of 100 ps.

### 2.12. Molecular Mechanics/Generalized Born Surface Area (MM/GBSA) Calculation

After the MD simulations, the binding free energies of the systems were calculated through the MM/GBSA model as implemented in the Prime MM/GBSA module in the Schrödinger Suite 2020-4. The OPLS3e force field, VSGB solvation model, and the default Prime parameters were used for the MM/GBSA calculations. The binding free energy was calculated for 10 frames based on the last 100 ns MD trajectory. The final frame of trajectories was analyzed after the MD simulations to compare and observe the structural deviation between wild-type and mutant structures of the SARS-CoV-2 spike RBD complexes.

## 3. Results and Discussion

Recently, a SARS-CoV-2 variant, B.1.351 (SA), that emerged seemed to increase the transmissibility of the infection and encode several mutations of RBD in the spike protein. In order to examine the effect of RBD mutations on viral infectivity, we first retrieved spike sequences according to B.1.351 and Wuhan lineages (reported in GISAID database). Indeed, D614G was found in all the variants, followed by the chosen additional six noteworthy amino acid changes (i.e., V367F, P384L, R408I, N501Y, K417N, E484K) in the spike RBD of both SARS-CoV-2 lineages. In particular, three mutations—K417N, E484K, and N501Y—appeared as particular mutation sites of B.1.351. Using site-directed mutagenesis and a full-length codon-optimized authentic SARS-CoV-2 spike (wild-type, WT) coding sequence [16], we constructed luciferase-expressing Maloney murine leukemia virus (MLV)-based pseudotyped virus bearing VSV glycoprotein (VSV-G) and several mutant spike proteins which represented Wuhan lineage (D614G, V367F/D614G, P384L/D614G, R408I/D614G) and SA lineage (N501Y/D615G, K417N/N501Y/D614G and E484K/N501Y/ D614G). 

**Figure 1:**
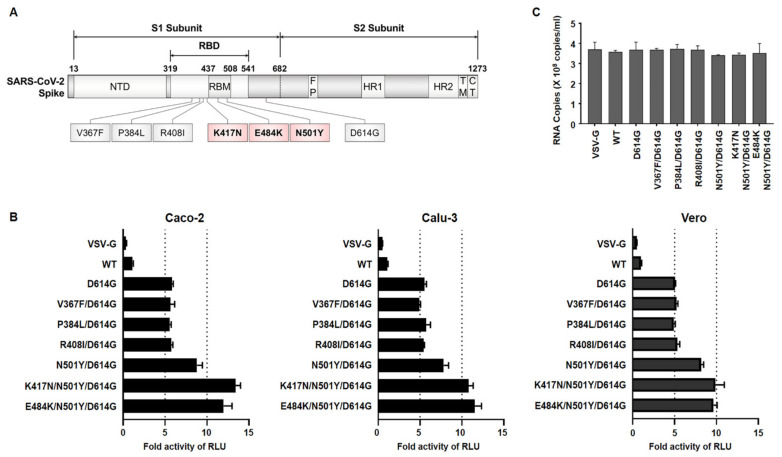
Infectivity of pseudoviruses harboring receptor-binding domain (RBD) mutants of spike protein either from Wuhan or B.1.351 lineages. (**A**) The illustration of amino acid changes selected RBD mutations for this study. Red-colored mutants represent the B.1.351 variant. Gray-colored mutants indicate the Wuhan variant. (**B**) Three different cell lines as indicated were infected with pseudoviruses with the Wuhan variant or South Africa variant. We also included VSV glycoprotein (VSV-G) protein-expressing pseudovirus as a negative control. The luminescence was measured after 24 h incubation and fold induction was calculated compared to SARS-CoV-2 Spike wild-type (WT). (**C**) The titer of each pseudovirus used in the experiment as shown in Figure 1B was quantified by RT-qPCR using primers targeting 3′ LTR region of the firefly luciferase reporter.

After the production of pseudoviruses, in order to determine their infectivity, three different cell lines were infected with the same particle numbers of pseudoviruses. We observed that three mutants regarded as B.1.351 (N501Y/D614G, K417N/N501Y/D614G, E484K/N501Y/D614G) infected these cells with approximately 2.5-fold higher than did D614G alone or in combination with the other variants (V367F/D614G, P384L/D614G, R408I/D614G) (Figure 1B). Due to the similarity among their pseudoviruses titer, the possibility of the observed enhanced infectivity being an artifact was excluded (Figure 1C). These data indicate that the mutations present within the RBM of RBD on the B.1.351 spike protein might induce the conformational change in the spike protein that leads to a higher binding affinity to ACE2, ultimately allowing for robust entry into the target cells compared with SARS-CoV-2 Wuhan lineage.

**Figure 2:**
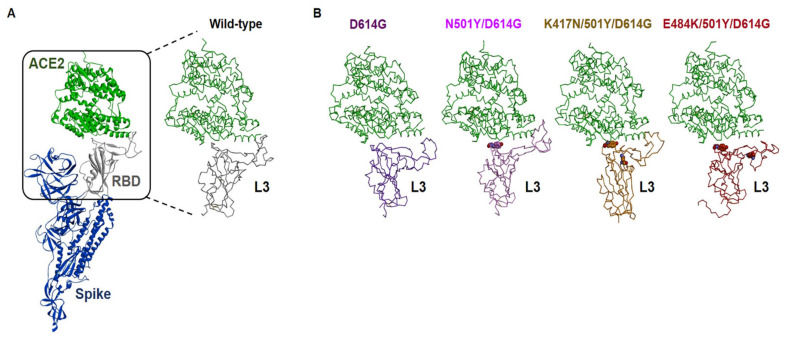
MD simulations of four mutants of the SARS-CoV-2 spike/ACE2 complex structures. (**A**) Homology model of building missing residues in the spike protein, and RBD binding interface of the wild-type spike protein. (**B**) Diverse conformational changes of flexible loop-3 (L3) region in the RBD of SARS-CoV-2 spike by MD simulations.

To examine the potential conformational changes due to the amino acid changes in the RBM in the spike protein of B.1.351 variant compared with WT and D614G variant of spike protein, we conducted 100 ns in silico Molecular Dynamic (MD) simulations for refinement of each monomeric mutant structure of SARS-CoV-2 spike protein complex with ACE2 residues 21-615, and trajectories obtained from MD simulations were used to calculate their binding affinities. Table 1 shows a comparison between the binding free energies (ΔG) of the spike proteins/ACE2 complexes for WT and mutant form. Three RBM mutants (K417N, E484K, and N501Y) together with D614G of spike protein showed higher binding affinity than WT. Notably, the E484K/N501Y/D614G mutant robustly increased binding affinity to ACE2 compared to either K417N/N501Y/D614G or N501Y/D614G. This increased affinity of K484 for ACE2 also correlated with large conformational change as shown in loop-3 (L3) in the RBM of the mutants (Figure 2 and Figure S1). This suggests that E484K mutant may have an impact on the stability of ACE2′s binding interface. Moreover, predicted ΔG values between mutant structures of SARS-CoV-2 spike and ACE2 values showed a correlation with the efficacy of infectivity as shown in Figure 1.

**Figure 3:**
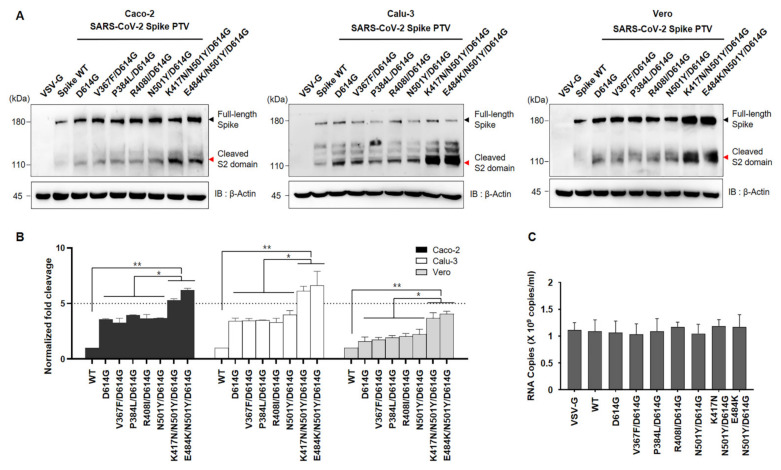
Effect of RBD mutation on spike cleavage (**A**) Murine leukemia virus (MLV) particles bearing VSV-G, SARS-CoV-2 spike (WT), SARS-CoV-2 Wuhan Spike (D614G, V367F/D614G, P384L/D614G, or R408I/D614G), or SARS-CoV-2 B.1.351 spike (N501Y, K417N/N501Y.D614G, or E484K/N501Y/D614G) were infected into indicated cell lines. Then cells were lysed and an equal amount of total proteins were analyzed by immunoblotting using an anti-SARS-CoV-2 Spike S2 domain antibody. (**B**) The measuring of band intensity of cleaved spike S2 domain (~100 kDa) and full-length spike (~180 kDa) was determined by semi-quantification of immunoblotting (IB), followed by calculated cleavage rate of each spike variants compared with Spike WT. (**C**) The titer of each pseudovirus used in the experiment as shown in Figure 3A was quantified by RT-qPCR. Data represent the mean ± SEM. Statistical analysis was conducted using a two-tailed Student’s *t*-test. *, *p* < 0.05; **, *p* < 0.005.

Given that the D614G has been shown to enhance cleavage efficiency due to substitution on spike conformational diversity [17,18], we next investigated the effect of RBD mutant of the spike on cleavage state of both Wuhan variant and SA variant. To do so, we transduced the seven different mutations of spike pseudoviruses into indicated cell lines namely Caco-2 and Calu-3 (ACE2+ and TMPRSS2+) or Vero (ACE2+), followed by determining the full length:S2 ratio by immunoblotting using an anti-spike antibody. Consistent with previous reports, we also observed that the D614G variant has more cleavage efficiency than WT as shown in Figure 3 bottom lane. In addition, the D614G combined with other residues show similar activity with D614G. Interestingly, the full length:S2 ratio is markedly greater in both K417N/N501Y/D614G and E484K/N501Y/ D614G compared to the other mutants including N501Y/D614G (Figure 3). Moreover, the total amount of the spike protein in pseudoviruses harboring both K417N/N501Y/D614G and E484K/N501Y/ D614G is also much higher than others, although the same titer of pseudovirus was transduced (Figure 3).

**Figure 4:**
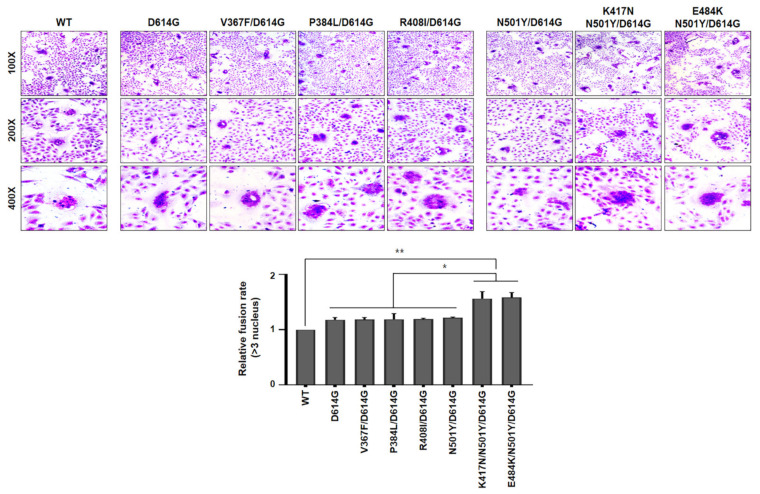
Cell-to-cell membrane fusion assay (syncytium formation assay) (Below) Vero cells were transfected with plasmids expressing each SARS-CoV-2 Spike RBD variant. At 24 h post-transfection, cells were treated with trypsin (1 μg/mL) before they were fixed, followed by staining with crystal violet. Representative images were captured at the indicated magnifications, and the number of syncytia containing more than three nuclei was counted. Fusion rate was calculated by dividing nuclei number in syncytia by total nuclei number (minimum 3000 total nuclei). The relative fusion rate was calculated by comparing SARS-CoV-2 Spike RBD mutants to WT and shown as a graph. Data represent the mean ± SEM. Statistical analysis was conducted using a two-tailed Student’s *t*-test. *, *p* < 0.05; **, *p* < 0.005.

These data further confirmed that the efficiency of B.1.351 SARS-CoV-2 lineage (particularly K417N/N501Y/D614G and E484K/N501Y/D614G) entry into target cells were greater than other variants. In order to ascertain whether cleavage capability of both K417N/N501Y/D614G and E484K/N501Y/D641G variants is required for cell-to-cell fusion, we tested the formation of syncytia. For this, Vero cells were transfected with each indicated spike variants, as shown in Figure 4. The spike (WT) of authentic SARS-CoV-2 was included as a control. At 24 h post-transfection, the cells were treated with trypsin, followed by staining with crystal violet. Interestingly, expression of spike either K417N/N501Y/D614G or E484K/N501Y/D614G variant significantly facilitated cell-to-cell fusion (syncytia formation) as compared to WT spike protein. Agreeing with cleavage assay in Figure 2, expression of several spike RBD mutants of Wuhan lineage marginally induced syncytial formation compared to WT spike protein (Figure 4). Taken together, our results indicate that B.1.351 lineage efficiently cleaved S1/S2 forms of spike protein than Wuhan lineage to promote cell-to-cell fusions, contributing to higher cell entry efficiency of B.1.351 SARS-CoV-2 than Wuhan SARS-CoV-2. However, the molecular mechanism by which spike mutant harboring either K417N/N501Y/D614G or E484K/N501Y/ D614G mutation promotes the cleavage efficacy of S1/S2 to facilitate membrane fusion activity needs to be further studied.

**Figure 5:**
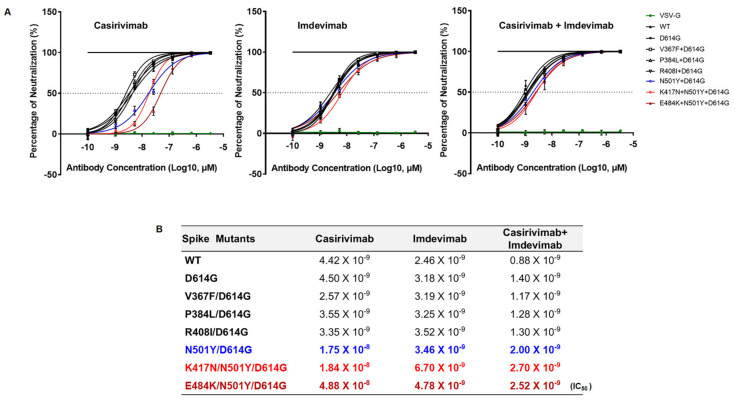
Neutralization assay of SARS-CoV-2 RBD targeting monoclonal antibody against spike RBD variants (**A**) Pseudoviruses expressing several indicated SARS-CoV-2 spike variants were incubated with two monoclonal antibodies (mAb) (Casirivimab and Imdevimab) with indicated concentration before transduced into 293T-ACE2 cells. After 24 h of incubation, the effect of SARS-CoV-2 spike mutation on mAb neutralization was analyzed by measuring the reduction of luciferase activity. The combination treatment was performed by treating half amount of both mAb. (**B**) IC50 values were calculated by GraphPad Prism 8 and shown for each variant. Red-colored mutants represent mutation from B.1.351 variant. The neutralization efficiency against VSV-G expressing pseudovirus was presented as a negative control for neutralization.

Since SARS-CoV-2 lineage B.1.351 emerged in the world with higher transmissibility, we examined the neutralizing activity of RBD-targeted two monoclonal antibodies, Casirivimab and Imdevimab (also known as REGN-CoV2) [15], against SARS-CoV-2 lineage B.1.351. All variants were comparably neutralized against SARS-CoV-2 lineage B.1.351 by Imdevimab, suggesting Imdevimab efficiently blocks the entry of the SA and Wuhan SARS-CoV-2 variants (Figure 5A). However, the spike variants (N501Y, K417N, E484K) harboring SARS-CoV-2 B.1.351 lineage was partially resistant against Casirivimab. We also examined neutralization escape after combination treatment of half-dose of Casirivimab and Imdevimab that bind distinct and non-overlapping regions of RBD (Figure 5B). Even though we utilized a half-dose of both monoclonal antibodies, we still saw a similar neutralization efficacy of Imdevimab against SARS-CoV-2 B.1.351 lineage as shown in single treatment (Figure 5B). Our data, therefore, suggest that combination treatment with different monoclonal antibodies which target non-overlapping RBD of the spike is a potentially powerful way to escape the emergence of novel variants of SARS-CoV-2.

As an obligate parasite, SARS-CoV-2 has continuously evolved over time in human populations, directly benefiting them with respect to their increased infectivity, transmissibility, and limiting their propagation. The emerging evidence raises concern about several new variants of SARS-CoV-2 with a potential increase in various mutations of the spike protein. These various mutations may provide an avenue for the SARS-CoV-2 to escape from immunity of the current vaccine against COVID19. Because of this, recent SARS-CoV-2 variant B.1.351, particularly those with mutations in the RBM of spike that potentially bring about conformational changes to the spike protein, got attention [1,17,19]. Herein, we show that K417N and E484K spike mutations derived from SARS-CoV-2 B.1.351 variant has a greater affinity for ACE2 as compared to the D614G derived from SARS-CoV-2 Wuhan variant via MD simulation-based predictions (Table 1 and Figure 2). Furthermore, both K417N and E484K substitution promote the cleavage of spike protein and cell-to-cell fusion, leading to enhanced SARS-CoV-2 infectivity (Figure 1, Figure 3 and Figure 4). We then examined whether these mutations are recognized by monoclonal antibodies, Imdevimab and Casirivimab. Our results found that neutralizing activity of Casirivimab, was significantly reduced against K417N/N501Y/D614G and E484K/N501Y/D614G mutations (Figure 5). Importantly, even with this reduced activity, Imdevimab that was tested was still capable to fully neutralize the pseudoviruses harboring K417N/N501Y/D614G and E484K/N501Y/D614G mutations (Figure 5). Taken together, our study clearly demonstrates that these spike changes promote SARS-CoV-2 antigenic drift, resulting in resistance to certain antibody neutralization. Moreover, this study demonstrates that K417N and E484K mutations are crucial in the enhancement of SARS-CoV-2 B.1.351 lineage infectivity and transmissibility. Further study is necessary to fully understand the biological relevance of these spike mutations in the context of SARS-CoV-2 genome. 

## Figures and Tables

**Table 1:** The binding free energies calculated from molecular dynamics (MD) simulations for the spike/ACE2 interactions of SARS-CoV-2.

	Average ΔG (kcal/mol)	Std. Dev.
Wild-type	−44.2180	17.95
D614G	−80.8294	14.41
N501Y/D614G	−87.0350	12.49
K417N/N501Y/D614G	−87.9461	19.21
E484K/N501Y/D614G	−121.4692	14.91

## Data Availability

The data presented in this study are available on request from the corresponding author.

## Supplementary Materials

The following are available online at https://www.mdpi.com/article/10.3390/v13040633/s1, Figure S1: The root mean square deviation (RMSD) analyses of MD simulations for mutant structure of SARS-CoV-2 spike complexed with ACE2.

## References

[1] Gribble J., Stevens L.J., Agostini M.L., Anderson-Daniels J., Chappell J.D., Lu X., Pruijssers A.J., Routh A.L., Denison M.R. (2021). The coronavirus proofreading exoribonuclease mediates extensive viral recombination. PLoS Pathog..

[2] Korber B., Fischer W.M., Gnanakaran S., Yoon H., Theiler J., Abfalterer W., Hengartner N., Giorgi E.E., Bhattacharya T., Foley B. (2020). Tracking Changes in SARS-CoV-2 Spike: Evidence that D614G Increases Infectivity of the COVID-19 Virus. Cell.

[3] Yurkovetskiy L., Wang X., Pascal K.E., Tomkins-Tinch C., Nyalile T., Wang Y., Baum A., Luban J. (2020). SARS-CoV-2 Spike protein variant D614G increases infectivity and retains sensitivity to antibodies that target the receptor binding domain. bioRxiv.

[4] Hou Y.J., Chiba S., Halfmann P., Ehre C., Kuroda M., Dinnon K.H., Leist S.R., Schäfer A., Nakajima N., Takahashi K. (2020). SARS-CoV-2 D614G variant exhibits efficient replication ex vivo and transmission in vivo. Science.

[5] Yurkovetskiy L., Wang X., Pascal K.E., Tomkins-Tinch C., Nyalile T.P., Wang Y., Baum A., Diehl W.E., Dauphin A., Carbone C. (2020). Structural and Functional Analysis of the D614G SARS-CoV-2 Spike Protein Variant. Cell.

[6] Mansbach R.A., Chakraborty S., Nguyen K., Montefiori D., Korber B., Gnanakaran S. (2020). The SARS-CoV-2 Spike Variant D614G Favors an Open Conformational State. bioRxiv.

[7] Volz E., Hill V., McCrone J.T., Price A., Jorgensen D., O’Toole Á., Southgate J., Johnson R., Jackson B., Nascimento F.F. (2021). Evaluating the Effects of SARS-CoV-2 Spike Mutation D614G on Transmissibility and Pathogenicity. Cell.

[8] Lee C.Y.-P., Amrun S.N., Chee R.S.-L., Goh Y.S., Mak T.-M., Octavia S., Ng L.F. (2020). Neutralizing antibodies from early cases of SARS-CoV-2 infection offer cross-protection against the SARS-CoV-2 D614G variant. bioRxiv.

[9] Muik A., Wallisch A.-K., Sänger B., Swanson K.A., Mühl J., Chen W., Cai H., Maurus D., Sarkar R., Türeci Ö. (2021). Neutralization of SARS-CoV-2 lineage B.1.1.7 pseudovirus by BNT162b2 vaccine–elicited human sera. Science.

[10] Kemp S.A., Meng B., Ferriera I.A., Datir R., Harvey W.T., Papa G., Lytras S., Colier D.A., Mohamed A., Gallo G. (2021). Recurrent emergence and transmission of a SARS-CoV-2 spike deletion H69/V70. bioRxiv.

[11] Teruel N., Mailhot O., Najmanovich R.J. (2021). Modelling conformational state dynamics and its role on infection for SARS-CoV-2 Spike protein variants. bioRxiv.

[12] Nie J., Li Q., Wu J., Zhao C., Hao H., Liu H., Zhang L., Nie L., Qin H., Wang M. (2020). Establishment and validation of a pseudovirus neutralization assay for SARS-CoV-2. Emerg. Microbes Infect..

[13] Carmo M., Peixoto C., Coroadinha A., Alves P., Cruz P., Carrondo M. (2004). Quantitation of MLV-based retroviral vectors using real-time RT-PCR. J. Virol. Methods.

[14] Hansen J., Baum A., Pascal K.E., Russo V., Giordano S., Wloga E., Fulton B.O., Yan Y., Koon K., Patel K. (2020). Studies in humanized mice and convalescent humans yield a SARS-CoV-2 antibody cocktail. Science.

[15] Baum A., Fulton B.O., Wloga E., Copin R., Pascal K.E., Russo V., Giordano S., Lanza K., Negron N., Ni M. (2020). Antibody cocktail to SARS-CoV-2 spike protein prevents rapid mutational escape seen with individual antibodies. Science.

[16] Shang J., Ye G., Shi K., Wan Y., Luo C., Aihara H., Geng Q., Auerbach A., Li F. (2020). Structural basis of receptor recognition by SARS-CoV-2. Nat. Cell Biol..

[17] Gobeil S.M.-C., Janowska K., McDowell S., Mansouri K., Parks R., Manne K., Stalls V., Kopp M.F., Henderson R., Edwards R.J. (2021). D614G Mutation Alters SARS-CoV-2 Spike Conformation and Enhances Protease Cleavage at the S1/S2 Junction. Cell Rep..

[18] Zhang L., Jackson C.B., Mou H., Ojha A., Peng H., Quinlan B.D., Rangarajan E.S., Pan A., Vanderheiden A., Suthar M.S. (2020). SARS-CoV-2 spike-protein D614G mutation increases virion spike density and infectivity. Nat. Commun..

[19] Benton D.J., Wrobel A.G., Roustan C., Borg A., Xu P., Martin S.R., Rosenthal P.B., Skehel J.J., Gamblin S.J. (2021). The effect of the D614G substitution on the structure of the spike glycoprotein of SARS-CoV-2. Proc. Natl. Acad. Sci. USA.

