# Supplementary material for: The Impact on Infectivity and Neutralization Efficiency of SARS-CoV-2 Lineage B.1.351 Pseudovirus
Source: Viruses. 2021 Apr 7;13(4):633. doi: 10.3390/v13040633 (PMC8067879; doi:10.3390/v13040633)

Supplemental Material

Supplemental Figure S1. The root mean square deviation (RMSD) analyses of MD simulations for mutant structure of SARS-CoV-2 spike complexed with ACE2. RMSD plots of protein backbone Cα carbon in (A) wild-type, (B) D614G, (C) N501Y:D641G, (D) K417N:N501Y:D641G, and (E) E484K:N501Y:D641G during the 100 ns of the MD simulations are depicted.

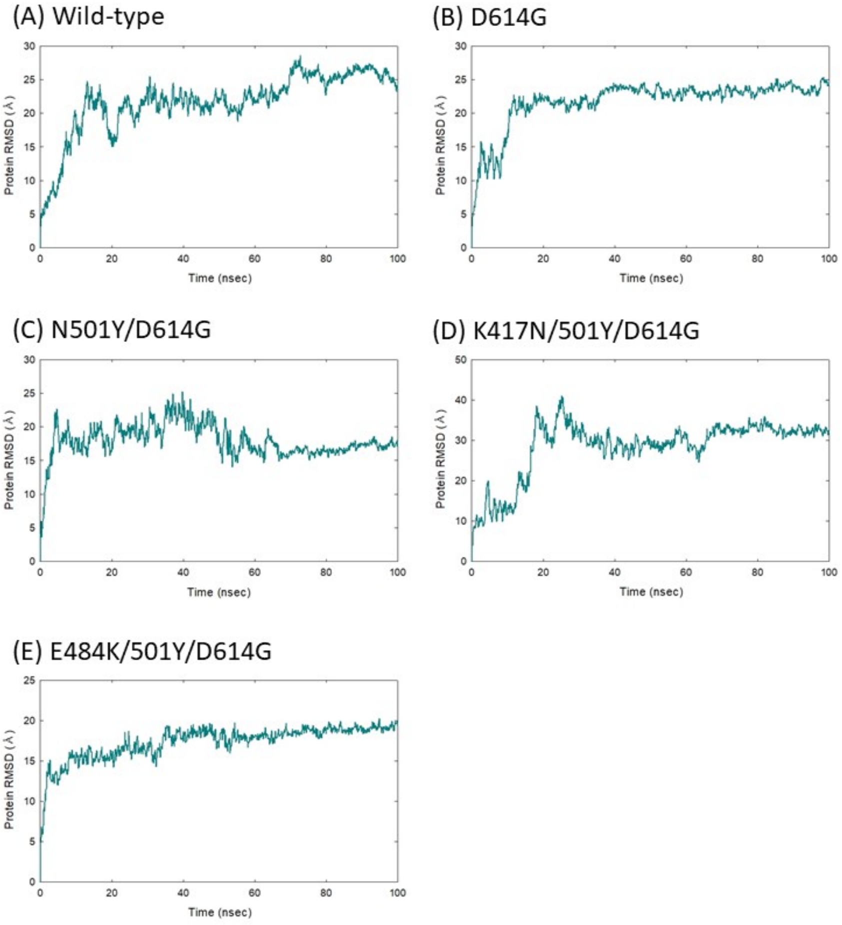

Supplement: Supplementary file 1 [file viruses-13-00633-s001.pdf]
